# Mechanical Properties and Biocompatibility of Urethane Acrylate-Based 3D-Printed Denture Base Resin

**DOI:** 10.3390/polym13050822

**Published:** 2021-03-08

**Authors:** Jy-Jiunn Tzeng, Tzu-Sen Yang, Wei-Fang Lee, Hsuan Chen, Hung-Ming Chang

**Affiliations:** 1 Ph.D. Program in Drug Discovery and Development Industry, College of Pharmacy, Taipei Medical University, Taipei City 110, Taiwan; d343105001@tmu.edu.tw; 2 Graduate Institute of Biomedical Optomechatronics, Taipei Medical University, No. 250, Wuxing St., Xinyi Dist., Taipei City 110, Taiwan; tsyang@tmu.edu.tw; 3 School of Dental Technology, Taipei Medical University, No. 250, Wuxing St., Xinyi Dist., Taipei City 110, Taiwan; weiwei@tmu.edu.tw; 4 National Yang Ming Chiao Tung University, No. 1001, University Road, Hsinchu 300, Taiwan; tess1994131@gmail.com; 5 Department of Anatomy and Cell Biology, Taipei Medical University, No. 250, Wuxing St., Xinyi Dist., Taipei City 110, Taiwan

**Keywords:** photopolymer resin, polyurethane acrylate, digital light processing, complete denture base

## Abstract

In this study, five urethane acrylates (UAs), namely aliphatic urethane hexa-acrylate (87A), aromatic urethane hexa-acrylate (88A), aliphatic UA (588), aliphatic urethane triacrylate diluted in 15% HDD (594), and high-functional aliphatic UA (5812), were selected to formulate five UA-based photopolymer resins for digital light processing (DLP)-based 3D printing. Each UA (40 wt%) was added and blended homogenously with ethoxylated pentaerythritol tetraacrylate (40 wt%), isobornyl acrylate (12 wt%), diphenyl (2,4,6-trimethylbenzoyl) phosphine oxide (3 wt%), and a pink acrylic (5 wt%). Each UA-based resin specimen was designed using CAD software and fabricated using a DLP 3D printer to specific dimensions. Characteristics, mechanical properties, and cytotoxicity levels of these designed UA-based resins were investigated and compared with a commercial 3D printing denture base acrylic resin (BB base) control group at different UV exposure times. Shore hardness-measurement data and MTT assays were analyzed using a one-way analysis of variance with Bonferroni’s post hoc test, whereas viscosity, maximum strength, and modulus were analyzed using the Kruskal–Wallis test (α = 0.05). UA-based photopolymer resins with tunable mechanical properties were successfully prepared by replacing the UA materials and the UV exposure times. After 15 min of UV exposure, the 5812 and 594 groups exhibited higher viscosities, whereas the 88A and 87A groups exhibited lower viscosities compared with the BB base group. Maximum flexural strength, flexural modulus, and Shore hardness values also revealed significant differences among materials (*p* < 0.001). Based on MTT assay results, the UA-based photopolymer resins were nontoxic. In the present study, mechanical properties of the designed photopolymer resins could be adjusted by changing the UA or UV exposure time, suggesting that aliphatic urethane acrylate has good potential for use in the design of printable resins for DLP-type 3D printing in dental applications.

## 1. Introduction

Complete denture prosthetics, including those made with acrylic denture base resins and artificial teeth, are among the main oral prosthetic devices used to replace a complete arch of missing teeth. Poly methyl methacrylate (PMMA) is the most commonly used resin in dentistry due to its low density, aesthetics, and cost-effectiveness [1], and can be used to fabricate a denture base through conventional processing techniques such as compression molding, fluid resin pouring, and injection molding [2]. However, maladaptation of dentures has been identified due to considerable polymerization shrinkage and feature distortion during processing [3,4].

With improvements in computer-aided design and computer-aided manufacturing (CAD-CAM) techniques, CAM-aided methods, such as a subtractive milling or additive three-dimensional (3D) printing processes, are alternatives to the conventional method of complete denture fabrication [5,6]. Although the recently introduced method of milling denture bases from prepolymerized PMMA blocks has reduced labor intensity and provided high machining accuracy, dentures with complex geometries such as those for undercuts or cavities remain difficult to manufacture [7].

Processes that involve 3D printing have evolved considerably and are well suited to fabricating complex structures directly from a digital design [8]. Among these, digital light processing (DLP), a liquid-based 3D printing technology, has recently become one of most popular methods used in dental applications due to its high printing resolution, fast production rate, and low costs [9]. DLP projectors use ultraviolet (UV) or visible light to induce a photopolymerization reaction that solidifies a photopolymer solution layer by layer [10]. Within dentistry, DLP-based 3D printers have been used to fabricate orthodontic models, surgical guides, crowns, bridges, dental splints, and denture bases [11].

**Figure 1:**
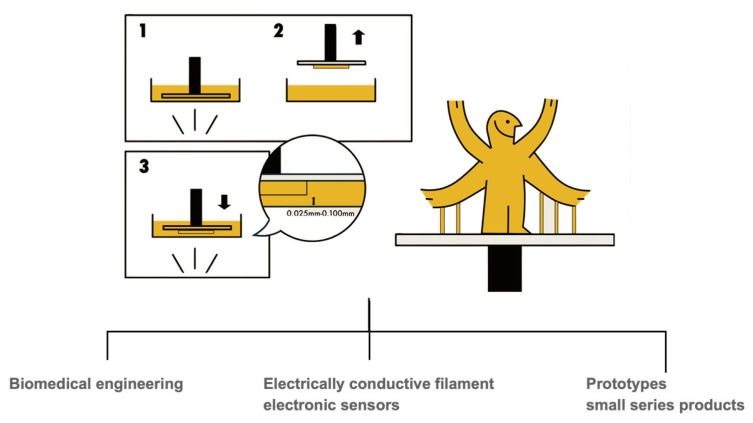
The application of acrylated polymers for additive manufacturing.

Studies of acrylated polymers for additive manufacturing have been extensive, examining conductivity, elasticity, and optical properties (Figure 1) [12]. Determining the formulation of photosensitive inks, which may contain acrylic oligomers, acrylic monomers, reactive diluents, and photoinitiators [13], is essential for exploiting DLP-based 3D printing in dental applications. Although various printable resins have been developed, several inherent limitations inhibit their use in clinical applications [14]. PMMA, for example, is a common light-curing resin used in the 3D printing industry. However, PMMA has a high shrinkage rate during light curing and poor mechanical properties [15]. Bisphenol A-glycidyl methacrylate (Bis-GMA) and urethane dimethacrylate (UDMA) are also used as light-polymerized dental composite resins; however, they have high molecular weights and viscosities [16]. Owing to their high wearability and inherent biocompatibility, polyurethanes have been widely used in biomedical devices such as dental aligners and artificial hearts [17,18]. However, printable urethane acrylate (UA)-based photopolymer resins with tunable mechanical properties for DLP-based 3D printers have been scarcely investigated and are rarely available for purchase.

The purpose of the present study was to develop potential composite resins with tunable mechanical properties for DLP-based 3D printers. Five UAs were chosen as monomers for photosensitive resins because they are well-established production materials [19]. Their characteristics, mechanical properties, and cytotoxicity levels were investigated.

## 2. Materials and Methods

### 2.1. Materials

Ethoxylated pentaerythritol tetraacrylate (PET5EO4A) and 5 UAs, namely aliphatic urethane hexa-acrylate (87A), aromatic urethane hexa-acrylate (88A), aliphatic UA (588), aliphatic urethane triacrylate diluted in 15% hexanediol diacrylate (HDDA) (594), and high-functional aliphatic UA (5812), were purchased from Double Bond Chemical Company (New Taipei City, Taiwan) and used as received. Diphenyl (2,4,6-trimethyl benzoyl) phosphine oxide (TPO, Sigma-Aldrich, Saint Louis, MO, USA) was employed as a photoinitiator and isobornyl acrylate (IBOA, Double Bond Chemical, New Taipei City, Taiwan) as a reactive diluent. For the control group, a commercial denture base acrylic resin for DLP-based 3D printers (BB base, Enlighten Materials, Taipei, Taiwan) was acquired.

### 2.2. Formulation of UA-Based Photopolymer Resins

Table 1 presents 5 formulas for printable UA-based photopolymer resins. One of the UAs, PET5EO4A (40 wt%), and a pink acrylic (5 wt%) were added to a glass beaker and blended to homogenity under a dark hood. Subsequently, the TPO initiator (3 wt%) and IBOA (12 wt%) were added to the mixture and magnetically stirred at 45 °C for 12 h to ensure homogeneity. Five designed resin solutions were then placed into high-density polyethylene tubes and stored in a dryer protected from light.

### 2.3. Specimen Fabrication

Specimens for all designed resin solutions used in the present study were designed using CAD software (DS SolidWorks, Waltham, MA, USA) to specific dimensions and exported as STL files. Files were subsequently processed with MiiUtility software (Young Optics, Hsinchu, Taiwan) to arrange specimen orientation on the build platform. A DLP 3D printer (MiiCraft 125, Young Optics, Hsinchu, Taiwan) was employed to cure the printable resins with a light-emitting diode projector (wavelength: 405 nm). Layers were sliced to a thickness of 50 μm, with an exposure time for each layer of 3 s.

After printing, specimens were soaked and magnetically stirred in a 95% ethanol bath for 5 min to remove unreacted resin. Specimens were then subjected to post-curing using Form Cure (Formlabs, MA, USA) with a wavelength of 405 nm at 60 °C. Specimens of each designed resin were divided into 3 experimental groups according to UV exposure time (0 min: T_0_, 15 min: T_15_, and 30 min: T_30_) to evaluate the effect of UV exposure on surface characterization.

### 2.4. Characterization

Plastic viscosity (η) of each designed resin was determined using a touch screen rheometer (DV3T, AMETEK Brookfield, Middleborough, MA, USA) with a V-74 spindle at room temperature. Mean η values were calculated using three measurements performed at a rotational speed of 5.5 rpm for 120 s.

Fourier transform infrared spectroscopy (FTIR, Nicolet iS5, Thermo Fisher Scientific, Waltham, MA, USA) in attenuated total reflection mode was used to determine the degree of double bond conversion (DC). Three specimens were fabricated with dimensions of 10 mm × 10 mm × 3 mm. DC was calculated using the following equation [17]:

$$DC\;\left(\%\right)=\frac{\left(\frac{C=C_{monomer}}{C=O_{monomer}}\right)-\left(\frac{C=C_{polymer}}{C=O_{polymer}}\right)}{\frac{C=C_{monomer}}{C=O_{monomer}}}\times 100\%$$

where $C=C_{monomer}$ and $C=C_{polymer}$ are absorbances at 810 cm^−1^ before and after exposure to UV light, respectively, and $C=O_{monomer}$ and $C=O_{polymer}$ are the absorbances within 1720–1730 cm^−1^ before and after exposure to the UV light, respectively [17].

For the three-point bending flexural test, 6 specimens of each designed resin (dimensions: 2 × 2 × 30 mm^3^) were fabricated and positioned on the center of 2 supporting rollers 3 mm in diameter and 20 mm apart. After that, a force was applied at the midpoint between supports using a universal testing machine (AGS-500G, Shimadzu, Kyoto, Japan) with a crosshead speed of 1 mm/min until breakage occurred. Maximum flexural strength was recorded and the flexural modulus were determined from the straight part of the stress-strain curve.

The Shore hardness of each designed resin was measured using a Shore durometer (Teclock GS-702G, Nagano Prefecture, Japan) according to ASTM D2240 [20]., Three measurements were performed on samples measuring 10 × 10 × 3 mm^3^ at each timepoint and mean values were calculated.

In vitro cytotoxic effects were evaluated using a 3-(4,5-dimethylthiazol-2-yl)-2,5-diphenyltetrazolium bromide (MTT) assay according to ISO 10993-5. At T_15_, three specimens of each designed resin with dimensions of 10 × 10 × 10 mm^3^ were soaked in Dulbecco’s modified Eagle’s medium for 24 h at 37 °C. Mouse fibroblast cells (L929) were cultured in minimum essential medium containing 10% fetal bovine serum (SBF) and 1% penicillin streptomycin for 24 h at 37 °C. The cells (5 × 10^4^ cell/well) were then seeded onto a 100-μL extract from the soaked specimen following ISO standards 10993012 in a 96-well plate. After 24 h of cell culturing, the media were removed and a 50-μL MTT solution was added. After cells were incubated for 4 h at 37 °C, optical density (OD) at 570 nm was measured using an ELISA reader (Multiskan, Thermo Fisher Scientific, Waltham, MA, USA). Cells cultured in a 96-well plate without the extract were saved for use as the negative control. Cell viability was calculated according to the following equation:

$$Viab.\;\left(\%\right)=\frac{OD_{s}-OD_{b}}{OD_{n}-OD_{b}}\times 100\%$$

where *s* represents the measured OD of the designed resin, and *n* and *b* represent the measured optical densities of the negative control and the blank, respectively.

### 2.5. Statistical Analysis

Statistical software (IBM SPSS Statistics, v19.0; Armonk, NY, USA, IBM Corp) was used for data analysis. After Kolmogorov–Smirnov goodness of fit and Levene’s tests, DC data, Shore hardness measurements, and MTT assays were compared among groups using one-way analysis of variance with Bonferroni’s post hoc test. Viscosity, maximum strength, and modulus were compared using the Kruskal–Wallis test (α = 0.05).

## 3. Results and Discussions

### 3.1. Viscosity

**Figure 2:**
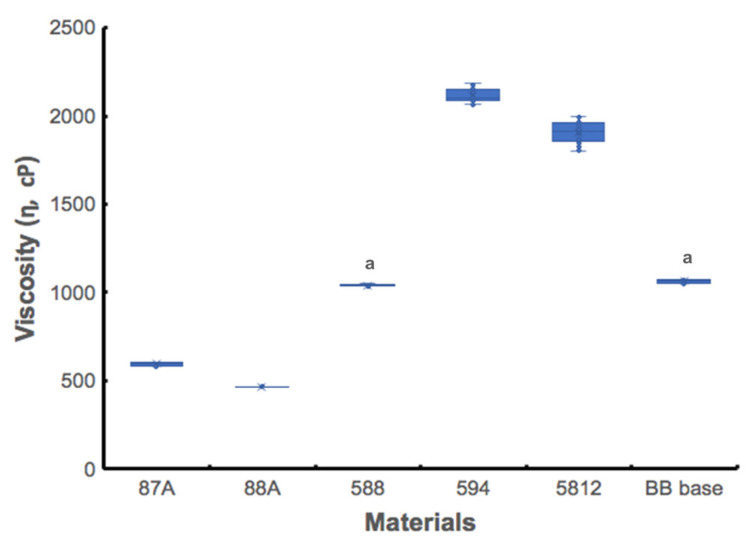
Viscosity of 5 UA-based photopolymer resins and a BB base. A same small letter indicates no static difference between materials.

Figure 2 shows viscosity test results, which reveal significant differences among materials (*p* < 0.001), listed here in ascending order: 88A, 87A, 588, BB base, 5812, and 594 groups. The low viscosity of printable resins is a critical parameter for preventing the formation of bubbles in 3D printed materials [16]. The 594 and 5812 groups had viscosities over 1000 cPs, meaning they were not suitable for 3D printing [19]. The 87A and 88A groups exhibited the lowest viscosities among the groups, and no significant difference was noted between the 588 and BB base groups.

### 3.2. FTIR Spectrum and Calculation of Degree of Conversion

**Figure 3:**
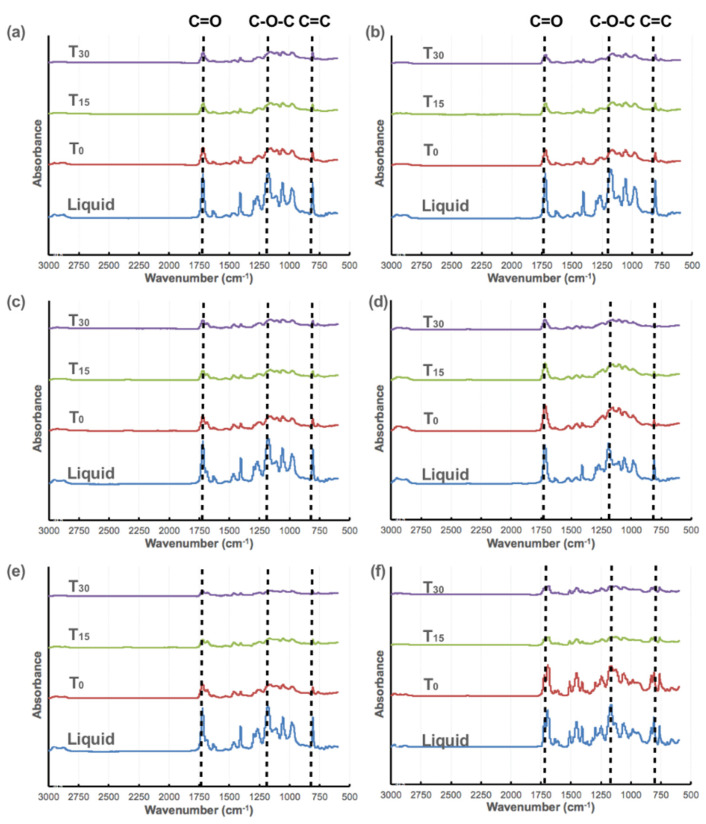
Fourier transform infrared spectrum of (**a**) 87A, (**b**) 88A, (**c**) 588, (**d**) 594, (**e**) 5812, and (**f**) a BB base.

Figure 3 shows the FTIR spectra of designed resins and the BB base control group. Strong absorption bands of the acrylate group (C=C) at 810 cm^−1^ and stretching vibrations of the (C=O) group at 1720 cm^−1^ were observed in the liquid state for all groups. The absorption features of the (CH_2_=CH) vinyl group at 985 cm^−1^, (C–O–C) stretching vibration at 1158 cm^−1^, (CH_2_=CH–R) scissoring at 1406 cm^−1^, and the asymmetric stretching vibrations and deformation of (C–H) in (–CH_2_–) and (–CH_3_) at 2860–2950 cm^−1^ were also presented [8]. The intensity of the absorption feature at 810 cm^−1^ weakened after the 3D printing process at T_0_, indicating that the C=C bonds gradually converted to saturate single bonds under UV light excitation. After post-curing processes, intensity decreased continuously and even disappeared at T_30_, as shown in Figure 3.

**Figure 4:**
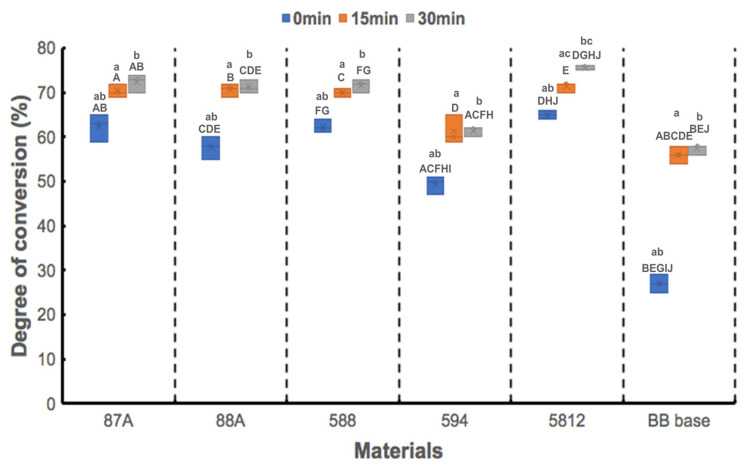
Degree of conversion for 5 UA-based photopolymer resins and a BB base. Same capital letter indicates static differences between materials for each UV-exposure period; same small letter indicates static difference between UV-exposure periods for each material.

The degree of C=C bond conversion was quantitatively calculated by reducing the peak intensity at 810 cm^−1^, as shown in Figure 4. At T_0_, the 5821 group had the highest DC of 68.14%, followed by the 588 and 87A groups, although these results did not reach statistical significance (*p* > 0.302). At 26.19%, the BB base group had the smallest DC among groups, reaching statistical significance (*p* < 0.008) at T_0_, T_15_, and T_30_, except for the 594 group at T_30_ (*p* = 0.07). There were no statistical differences in DC among groups (*p* > 0.054), excepting the BB group (*p* < 0.008) at T_15_. Significant improvements in DC of each material occurred after UV curing for 15 min (*p* < 0.016) and 30 min (*p* < 0.005); however, no statistical differences were observed between T_15_ and T_30_, except for the 5812 group (*p* = 0.004).

In the present study, the carbonyl group bond at 1720 cm^−1^ was considered the standard internal peak due to its high stability [19]. The degree of C=C bond conversion is a crucial index for assessing the photocuring reactivity of photopolymer resins; this provides information on selection parameters of the photoinitiator. The lowest DC was observed in the BB base group at T_0_, suggesting the presence of a large amount of unreacted double bonds [21]. Unreacted double bonds and photoinduced free radicals or surface oxygen around the forming 3D network structure may hinder further polymerization [22]. Thus, post-curing was recommended [23]. All groups’ DC increased concomitantly with UV exposure time, a finding in line with a previous study [17].

### 3.3. Mechanical Properties

**Figure 5:**
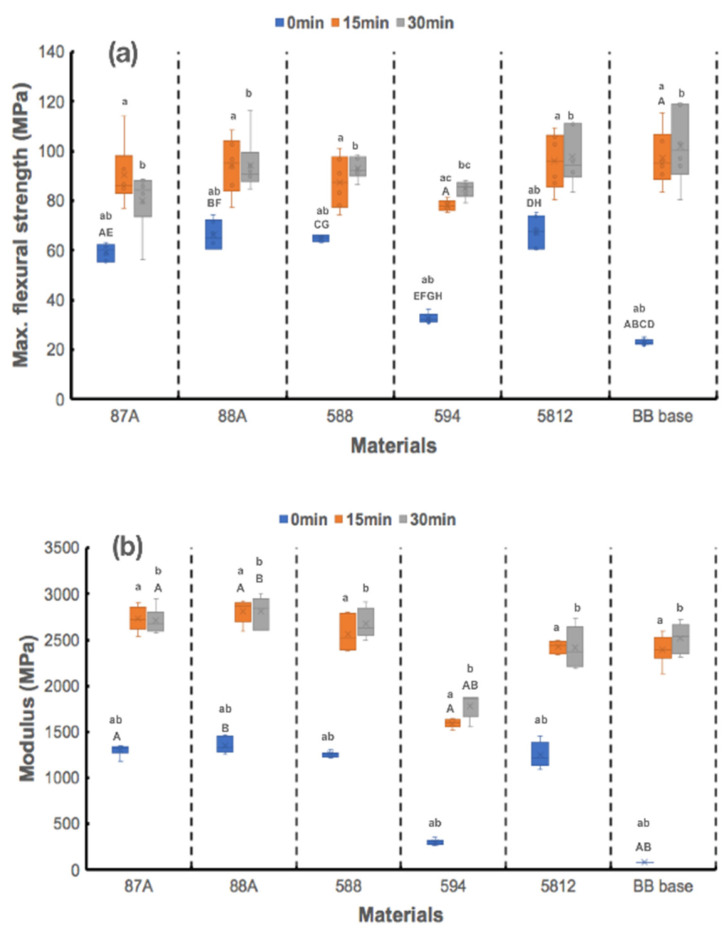
(**a**) Flexural strength and (**b**) flexural modulus of 5 urethane acrylate (UA)-based photopolymer resins and a BB base. Same capital letter indicates static difference between materials for each UV-exposure period; same small letter indicates static difference between UV-exposure periods for each material.

Mechanical properties were evaluated using the three-point bending flexural test and Shore hardness measurements. Figure 5 shows the results of maximum flexural strength and modulus. As shown in Figure 5a, statistically significant differences in maximum flexural strength were found among the printed materials at T_0_ (*p* < 0.001) and T_15_ (*p =* 0.023); however, no statistical differences were observed at T_30_ (*p =* 0.093). Maximum flexural strengths of the designed resin groups were all significantly higher than the BB base group at T_0_, excepting the 594 group (*p =* 0.454). After 15 min of UV curing, the 594 group exhibited the smallest maximum flexural strength, but a statistical difference was only observed between the BB base and 594 groups (*p =* 0.032). UV exposure time significantly influenced the maximum flexural strengths of the designed resin groups (*p* < 0.001 for the 87A, 88A and 594 groups; *p* = 0.003 for the 588 and BB base groups), except for the 5812 group (*p* = 0.752), revealing that 15 min of UV curing significantly improved the bending performance of these printed resins (*p* = 0.022 for the BB base group; *p* = 0.03 for the 588 group; *p* < 0.001 for the 87A, 88A and 594 groups). A statistical difference in maximum flexural strengths was only observed in the 594 group between T_15_ and T_30_ (*p* = 0.002).

As shown in Figure 5b, statistically significant differences in flexural modulus were observed among printed materials at T_0_, T_15_, and T_30_ (all *p <* 0.001). At T_0_, the BB base group exhibited smaller flexural modulus than the 87A (*p =* 0.003) and 88A (*p <* 0.001) groups. The 594 group exhibited the smallest flexural modulus among materials both at T_15_ (*p <* 0.001 as compared with 88A) and T_30_ (*p =* 0.007 as compared with 87A; *p =* 0.024 as compared with 588). UV exposure time also significantly influenced flexural modulus of the designed resin groups (*p =* 0.003 for the 87A, 88A, 588, and BB base groups; *p =* 0.001 for the 594 group); however no statistical differences were observed for any materials between T_15_ and T_30_ (all *p* > 0.05).

**Figure 6:**
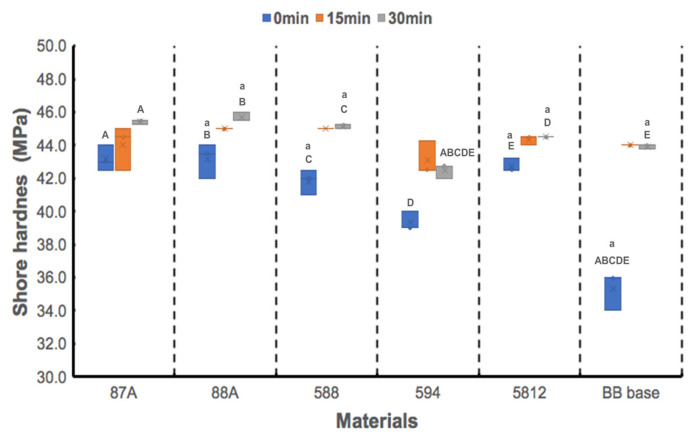
Shore hardness values of 5 urethane acrylate UA-based photopolymer resins and a BB base. Same capital letter indicates static difference between materials for each UV-exposure period; same small letter indicates static difference between UV-exposure periods for each material.

Figure 6 shows hardness values of the printed resins, revealing statistically significant differences among the printed materials at T_0_ (*p* = 0.016), T_15_ (*p* = 0.031), and T_30_ (*p* = 0.006). All designed resin groups exhibited significantly higher surface hardness than the BB base group at T_0_ (all *p* < 0.001). After 30 min of UV exposure, the surface hardness of the 594 group was the lowest among groups (all *p* < 0.001). UV exposure time significantly influenced surface hardness of the 88A (*p* = 0.023), 588 (*p* = 0.033), 5812 (*p* = 0.034), and BB base (*p* = 0.034) groups. Statistical differences in surface hardness were observed between T_0_ and T_30_ for the 88A (*p* = 0.018), 588 (*p* = 0.03), 5812 (*p* = 0.014), and BB base (*p* = 0.041) groups; however, no statistical differences were noted between T_15_ and T_30_ for these groups (all *p* > 0.05).

In the present study, four types of aliphatic urethane acrylates and one type of aromatic urethane acrylate were used. The urethane acrylate oligomers, which consisted of either aromatic or aliphatic types, provided the proper balance of competing properties, such as flexibility, resistance to discoloration, chemical resistance, and cure rate [24,25]. The aromatic type offered a balance between reactivity, toughness, and hardness, whereas the aliphatic type had good color stability and durability. The saturated alkanes and cycloalkanes are the main chin of the 588 group, resulting in comparable mechanical properties (mean max. strength: 92.45 MPa, mean modulus: 2.56 GPa, mean shore hardness: 45 HA) at T_15_ and viscosity (1040.55 cPs) at T_0_ to the commercially branded BB-based group (mean max. strength: 97.25 MPa; mean modulus: 2.40 GPa; mean shore hardness: 44 HA; mean viscosity: 1062.96 cPs).

### 3.4. Cytotoxicity Test

**Figure 7:**
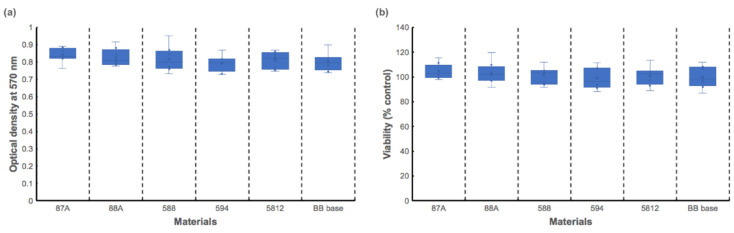
MTT assay results showing L929 cell viability after incubation in extracts from 5 UA-based photopolymer resins and a BB base. (**a**) optical density values, (**b**) viability.

To ensure the designed resin extracts were nontoxic, a cytotoxicity test was performed. MTT assay results for L929 cells cultured in of the printed resin extracts are shown in Figure 7. The 87A group had the highest OD value, whereas the 594 group exhibited the lowest OD value (Figure 7a). However, no statistical differences were found among the groups (*p* = 0.764). Also, Figure 7b shows relative viabilities of all groups above 70%, indicating that these printed resins are nontoxic [16]. Residual organic solvent, leaching of unreacted monomer, degradation products, and photoinitiators are potential sources of cytotoxic damage [26]. MTT assay results in the present study suggest that the above-mentioned sources of cytotoxicity were negligible.

A limitation of the present study exists in that only one UA PET5EO4A ratio was used in formula calculation. Although the aliphatic urethane acrylates 87A and 588 exhibited mechanical properties comparable to commercial products, the optimal ratio of UA to PET5EO4A has yet to be determined. This limitation should be addressed in future studies.

## 4. Conclusions

UA-based photopolymer resins were successfully synthesized. Specimens printed using a DLP-type printer containing 40% aliphatic UA (that is, the 588 group) exhibited high printability. Further post-curing for 15 min improved specimens’ mechanical properties. These properties are comparable with commercial products, suggesting that aliphatic urethane acrylate has promising potential use as a printable resin material in DLP-type 3D printing, and high potential commercial value.

## Figures and Tables

**Table 1:** Preparative percentages (wt%) of 5 urethane acrylate-based photopolymer resins.

Group Name	Monomer				
	UA	PET5EO4A	TPO	IBOA	Acrylic
87A	40% aliphatic urethane hexa-acrylate	40	3	12	5
88A	40% aromatic urethane hexa-acrylate	40	3	12	5
588	40% aliphatic urethane acrylate	40	3	12	5
594	40% aliphatic urethane triacrylate diluted in 15% HDDA	40	3	12	5
5812	40% high functional aliphatic urethane acrylate	40	3	12	5

PET5EO4A: Ethoxylated pentaerythritol tetraacrylate; TPO: Diphenyl (2,4,6-trimethylbenzoyl) phosphine oxide; IBOA: isobornyl acrylate.

## Data Availability

The data presented in this study are available on request from the corresponding author.
